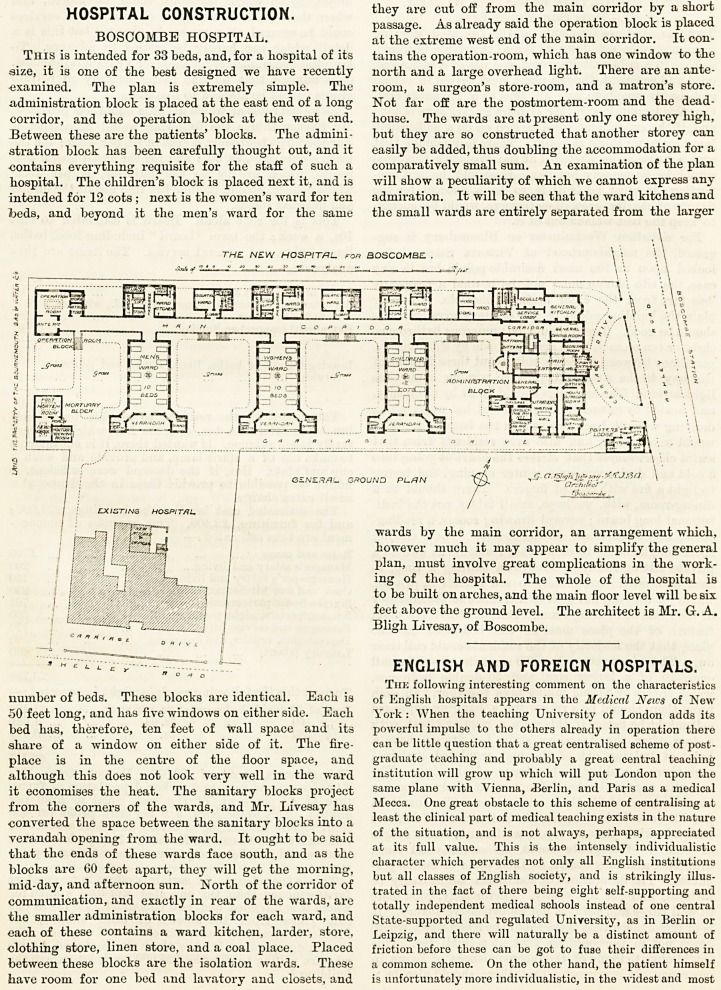# Hospital Construction

**Published:** 1899-11-25

**Authors:** 


### THE INSTITUTIONAL WORKSHOP.

### HOSPITAL CONSTRUCTION.

### BOSCOMBE HOSPITAL.

THIS is intended for 33 beds, and, for a hospital of its size, it is one of the best designed we have recently examined. The plan is extremely simple. The administration block is placed at the east end of a long corridor, and the operation block at the west end. Between these are the patients' blocks. The administration block has been carefully thought out, and it contains everything requisite for the staff of such a hospital. The children's block is placed next it, and is intended for 12 cots; next is the women's ward for ten beds, and beyond it the men's ward for the same number of beds. These blocks are identical. Each is 50 feet long, and has five windows on either side. Each bed has, therefore, ten feet of wall space and its share of a window on either side of it. The fireplace is in the centre of the floor space, and although this does not look very well in the ward it economises the heat. The sanitary blocks project from the corners of the wards, and Mr. Livesay has converted the space between the sanitary blocks into a verandah opening from the ward. It ought to be said that the ends of these wards face south, and as the blocks are 60 feet apart, they will get the morning, mid-day, and afternoon sun. North of the corridor of communication, and exactly in rear of the wards, are the smaller administration blocks for each ward, and each of these contains a ward kitchen, larder, store, clothing store, linen store, and a coal place. Placed between these blocks are the isolation wards. These have room for one bed and lavatory and closets, and they are cut off from the main corridor by a short passage. As already said the operation block is placed at the extreme west end of the main corridor. It contains the operation-room, which has one window to the north and a large overhead light. There are an ante-room, a surgeon's store-room, and a matron's store. Not far off are the postmortem-room and the dead-house. The wards are at present only one storey high, but they are so constructed that another storey can easily be added, thus doubling the accommodation for a comparatively small sum. An examination of the plan will show a peculiarity of which we cannot express any admiration. It will be seen that the ward kitchens and the small wards are entirely separated from the larger wards by the main corridor, an arrangement which, however much it may appear to simplify the general plan, must involve great complications in the working of the hospital. The whole of the hospital is to be built on arches, and the main floor level will be six feet above the ground level. The architect is Mr. G. A. Bligh Livesay, of Boscombe.

## Figures and Tables

**Figure f1:**